# Rethinking Naturalistic Movie Neuroimaging Through Film Form

**DOI:** 10.3390/bs16050639

**Published:** 2026-04-24

**Authors:** Zhengcao Cao, Yashu Wang, Xiang Xiao, Yiwen Wang

**Affiliations:** 1 School of Arts and Communication, Beijing Normal University, Beijing 100875, China; 2 Faculty of Arts and Sciences, Beijing Normal University, Zhuhai 519087, China

**Keywords:** naturalistic movie neuroimaging, film forms, mediating layer, cognitive processing, film cognition matrix

## Abstract

Understanding how the brain processes complex real-world experiences remains a central challenge in cognitive neuroscience. Naturalistic movie neuroimaging has gained prominence by using temporally continuous stimuli that approximate everyday perception. However, cinematic experience is not equivalent to real-world cognition. Films are systematically constructed through film forms such as editing, camera movement, and sound, which diverge from natural perceptual conditions and shape cognitive processing. In this review, we rethink naturalistic movie neuroimaging by foregrounding film form as a central explanatory factor. We propose a conceptual framework for studying human cognition through film form, in which film form is conceptualized as a mediating layer between naturalistic movie neuroimaging and cognitive processing. We synthesize behavioral and neuroimaging evidence showing that multiple film forms exert domain-specific influences on attention, emotion, and memory. To organize these findings, we propose the Film Cognition Matrix, which maps film forms onto core cognitive domains and supports comparative research. Finally, we argue that interpretations of naturalistic movie neuroimaging should explicitly model film form as a mediator. Future directions include computationally modeling to isolate film-form effects on neural activity, expanding film-form–cognition mapping, exploring interactive and immersive media, and clarifying the boundary between real-world cognition and cinematic aesthetics.

## 1. Introduction

The central aim of cognitive research is to understand how humans perceive, interpret, and respond to information in the real world. Traditionally, cognitive neuroscience has relied on highly controlled experimental paradigms that simplify stimuli and tightly manipulate variables (Cohen et al., 2017; LaBar & Cabeza, 2006). While indispensable for mechanistic decomposition and causal inference, such paradigms often remove cognition from the complex and natural contexts in which it unfolds (Maren et al., 2013; Oliva & Torralba, 2007). As a result, they provide limited leverage for capturing cognition as a dynamic, continuous, and socially embedded process (Lloyd & Leslie, 2013; Nikolić, 2010). Against this background, research on naturalistic cognition has gained momentum, aiming to bridge the gap between laboratory tasks and real-world cognitive processes (Hasson et al., 2008; Saarimaeki, 2021).

Among various naturalistic approaches, movie-based neuroimaging has attracted particular attention. Film is a temporally continuous and multimodal audiovisual medium, and it elicits rich cognitive and emotional responses under controlled conditions (Hasson et al., 2004; Hasson et al., 2010b). Movie viewing requires the brain to continuously integrate and predict information over time, a process that closely parallels everyday perceptual processing (Hasson et al., 2008). Compared with trial-based stimuli, films more closely approximate continuous human perceptual environments (Bartels & Zeki, 2004; Nastase et al., 2019; Sonkusare et al., 2019). Moreover, through shooting and editing, films partially simulate the sampling mechanisms of the human visual system (Cutting, 2015; T. J. Smith, 2012; T. J. Smith & Mital, 2013). These film properties make movie-based paradigms well-suited for examining temporally extended cognitive processes, including attention, emotion, narrative comprehension, and social cognition (Cao et al., 2026; Hasson et al., 2008; Jääskeläinen et al., 2021; Redcay & Moraczewski, 2020; Saarimaeki, 2021; Zacks et al., 2010). Accordingly, naturalistic movie neuroimaging has become an influential methodological approach for investigating temporal brain dynamics and inter-subject synchronization, as well as higher-order cognition and clinical applications (Cha et al., 2015; Eickhoff et al., 2020; Hasson et al., 2010a; Hasson & Honey, 2012; Lahner et al., 2024; Meer et al., 2020).

However, when movies are used to approximate real-world experience, their film-form characteristics cannot be ignored (Sonkusare et al., 2019). Films are not passive recordings of reality but highly structured audiovisual media that systematically manipulate time and space through film form (Bordwell, 1985; Bordwell & Thompson, 2008; G. M. Smith, 2003). Editing reorganizes temporal structure; camera movement constrains spatial perception; and color and sound guide attention and emotional experience. These film forms actively shape cognitive processing rather than merely decorating narrative content (Hasson et al., 2008; Tan, 2018). Behavioral and physiological studies have demonstrated that movie viewing induces unusually strong attentional engagement and reduced spontaneous blinking (Andreu-Sánchez et al., 2017; Martín-Pascual et al., 2018), synchronized gaze patterns (Hinde et al., 2018; Loschky et al., 2015; T. J. Smith & Mital, 2013), as well as altered emotional perception produced through structured face–context shot combinations, as illustrated by the Kuleshov effect (Bordwell, 1972; Cao et al., 2024b; Kuleshov & Kuleshov, 1974). Compared with real-world perception, perceptual activity during movie viewing is largely shaped by directors and editors, resulting in reduced perceptual autonomy alongside enhanced attentional focus, emotional intensity, and inter-subject synchrony (Grall & Finn, 2022; Sonkusare et al., 2019). Because movie viewing combines ecological richness with formal constraint, cognition elicited by film simultaneously exhibits properties of real-world naturalistic cognition and distinct “cinematized” characteristics. It is precisely this dual nature that gives rise to a central interpretive challenge in movie-based neuroimaging (Grall & Finn, 2022; Sonkusare et al., 2019). In addition, many studies analyze brain activity during movie viewing by treating films as holistic stimuli, focusing on large-scale neural patterns or inter-subject synchronization (Baldassano et al., 2018; Rajimehr et al., 2024; Raz et al., 2016), while paying limited attention to the specific roles of individual film forms. Therefore, when naturalistic movie neuroimaging is used as a research tool, its specificity as film form should be explicitly considered, as many studies have shown that editing, camera movement, and color systematically affect attention and emotion (Calbi et al., 2019; Cao et al., 2024a; Heimann et al., 2019; Kolesnikov et al., 2025; T. J. Smith, 2012). Only by accounting for film form and its modulatory effects on cognition can data obtained from movie neuroimaging be interpreted with greater precision and theoretical depth.

In this review, we aim to synthesize findings from naturalistic movie neuroimaging and systematically delineate how film form shapes cognitive processing. First, we clarify that real-world cognition is the ultimate goal of inquiry, and that naturalistic movie neuroimaging represents a methodological step toward this goal. Building on this premise, we propose a conceptual framework for studying human cognition through film form, distinguishing naturalistic movie neuroimaging as a research paradigm from film form as a mediating mechanism between movie stimuli and cognition. Within this framework, we organize accumulated evidence into the Film Cognition Matrix that links distinct film forms (e.g., editing, camera movement, color) to core cognitive domains (e.g., attention, emotion, memory). Finally, we outline future directions for advancing more interpretable and theoretically grounded naturalistic movie neuroimaging research.

## 2. Current Perspectives on Naturalistic Movie Neuroimaging

Naturalistic movie neuroimaging studies have shown that movie viewing reliably engages neural systems supporting emotion, memory, and social cognition. In this section, we first revisit representative findings and analytical approaches, then clarify the interpretive limitations and how film form is embedded in existing studies and analytical approaches.

### 2.1. Representative Findings

Across cognitive domains, naturalistic movie paradigms have yielded robust evidence that movie viewing engages distributed and dynamic neural systems supporting emotion, memory, and social cognition. Specifically, in emotion research, movie viewing reliably elicits rich and evolving affective experiences, recruiting networks including the amygdala, insula, orbitofrontal cortex, and default-mode network (Cahill et al., 1996; Kragel & LaBar, 2015; Saarimäki et al., 2016; Westermann et al., 1996). Multivariate analyses further demonstrate that both basic and complex social emotions can be differentiated based on spatial activity patterns (Saarimäki et al., 2016, 2018), while temporally resolved analyses reveal sequential patterns of activation that unfold across sensory, limbic, and midline regions during emotionally engaging scenes (Pujol et al., 2018; Sabatinelli et al., 2006). Beyond emotion, memory research using movies shows that encoding and segmentation of continuous experience depend on coordinated activity among the hippocampus, medial prefrontal cortex, and default-mode network (J. Chen et al., 2016; Hasson et al., 2008; van Kesteren et al., 2010), with event boundaries reliably eliciting hippocampal responses predictive of later recall (Baldassano et al., 2017; Ben-Yakov & Dudai, 2011). Importantly, many such boundaries are introduced through cinematic segmentation, including cuts, transitions, and pacing, rather than reflecting intrinsic properties of real-world events. This pattern suggests that film form operates as a structuring mechanism for memory encoding, even when such findings are framed in terms of cognitive event parsing (J. Chen et al., 2017; Milivojevic et al., 2015). Similarly, naturalistic movie stimuli have expanded the study of social cognition by capturing extended and contextually embedded social interactions. Such movie paradigms consistently implicate the default-mode network, temporoparietal junction, posterior superior temporal sulcus, and medial prefrontal cortex in mentalizing and perspective-taking during social comprehension (Iacoboni et al., 2004; Lahnakoski et al., 2012; Yeshurun et al., 2017). Unlike everyday social cognition, access to characters’ mental states during movie viewing is systematically structured by cinematic perspective. Framing and temporal disclosure shape how viewers infer intentions and emotions (Bacha-Trams et al., 2017; Grall & Finn, 2022). Taken together, across emotion, memory, and social cognition, neural responses elicited by naturalistic movie viewing are predominantly interpreted in terms of narrative meaning. By contrast, film forms such as editing, camera movement, sound design, and visual framing are relegated to a secondary role, despite their systematic capacity to structure cognitive processing over time (Andreu-Sánchez et al., 2017; Hinde et al., 2018; Shimamura et al., 2013; T. J. Smith & Mital, 2013).

### 2.2. Analytical Approaches and Interpretive Limitations

At the analytical level, current approaches in naturalistic movie neuroimaging have demonstrated that movie viewing elicits reliable, synchronized neural activity across viewers (Gao et al., 2020; Hasson et al., 2010b; Meer et al., 2020). However, these approaches are not optimized to examine film form as a mediating variable in cognition. Approaches such as inter-subject correlation (ISC) and representational similarity analysis (RSA) were developed to accommodate continuous and multimodal naturalistic stimuli by capturing shared brain dynamics across individuals (Jääskeläinen et al., 2021; Nastase et al., 2019; Redcay & Moraczewski, 2020). Their primary goal is to quantify the temporal alignment of neural responses, rather than to isolate the causal contribution of specific stimulus structures. Accordingly, films are typically modeled as unitary stimuli (Vanderwal et al., 2019). Related approaches, such as event-based models, privilege narrative or semantic annotations (Baldassano et al., 2017; Kauttonen et al., 2015), while network analyses characterize large-scale integration without explicitly modeling film forms (Mochalski et al., 2024). ISC and RSA thus abstract neural activity into population-level synchrony (Hasson et al., 2008; Jääskeläinen et al., 2021), rendering the compositional elements that guide attention and perception analytically opaque. A key limitation of this abstraction is that film form is inherently multivariate and systematically covaries with narrative content, as changes in editing, sound, and narrative emphasis rarely occur in isolation. When multiple film forms covary over time, high-level outcome measures such as neural synchrony or network reconfiguration become difficult to interpret, making it challenging to attribute observed effects to narrative comprehension, attentional guidance, affective modulation, or their interactions (Kauttonen et al., 2015). As a result, film form is absorbed into aggregate neural signatures rather than modeled as an explanatory mediator. This limitation is reinforced by recent large-scale evidence. Leipold et al. (Leipold et al., 2025) demonstrated substantial between-movie variability in ISC and brain–behavior associations across animated films, indicating that neural synchronization is strongly stimulus-dependent rather than a generic property of movie viewing. Rather than supporting broad generalizations about naturalistic cognition, these findings highlight a mismatch between analytical models that assume functional equivalence across movies and stimuli whose cognitive effects are shaped by distinct film forms (Leipold et al., 2025; Sonkusare et al., 2019). Taken together, this body of work suggests that a central limitation of current naturalistic movie neuroimaging lies not only in stimulus complexity but in analytical frameworks that describe shared neural responses without specifying how film form shapes cognitive processing, leaving its role insufficiently theorized.

## 3. Film Form as a Mediator of Cognition in Naturalistic Movie Neuroimaging

Through the review in the previous section, film form is shown to be embedded in naturalistic movie neuroimaging studies and analytical approaches aimed at investigating real-world cognition. This section introduces a conceptual framework for examining cognition through film form, synthesizes empirical evidence supporting its mediating role, and proposes the Film Cognition Matrix to organize existing findings and guide future research.

### 3.1. A Framework for Studying Human Cognition Through Film Form

Rather than treating movies primarily as approximations of real-world experience, this framework begins from an asymmetry between everyday perception and cinematic cognition. In everyday perception, cognitive processing unfolds through self-directed exploration guided by endogenous attention and continuous multisensory feedback, whereas cinematic experience is externally structured by design. In addition, movies capture fragments of reality and depict everyday social situations, an ecological richness that has made them valuable tools for studying complex phenomena such as emotion and social understanding in neuroimaging (Astolfi et al., 2009; Hanke et al., 2014; Saarimäki et al., 2016; Spiers & Maguire, 2007). However, it should be noted that real-world cognition is the ultimate goal of inquiry, and films should therefore be understood as an experimentally tractable yet constrained methodological means rather than an end in themselves. Films are subjective and goal-directed constructions, designed to inform or entertain. In this process, naturalness is selectively shaped to serve communicative and aesthetic aims (Johnson-Sheehan & Paine, 2015; Lasswell, 1948). Accordingly, formal choices in editing, camera movement, lighting, and sound systematically guide viewers’ attention, emotion, and interpretation, shifting information sampling from self-guided exploration to externally structured modes. This externally structured mode of engagement is reflected in empirical findings. For example, spontaneous blink rates are reduced during screen viewing relative to real-world observation (Andreu-Sánchez et al., 2017; Martín-Pascual et al., 2018). In addition, stronger Mu-band event-related desynchronization is observed in sensorimotor cortices during real-world action observation than during on-screen viewing, suggesting a more embodied mode of perceptual engagement in real-world contexts (Andreu-Sánchez et al., 2024). Taken together, these features place cinematic stimuli between real-world experience and laboratory tasks: movies preserve key aspects of naturalistic perception while embedding them within a structured, film-form aesthetic regime. From this perspective, film form functions not as a passive carrier of narrative meaning but as a mediating layer that organizes sensory, emotional, and narrative information before and during cognitive processing, underscoring the need for a framework that treats neural responses during movie viewing as mediated rather than direct engagement with real-world information.

**Figure 1:**
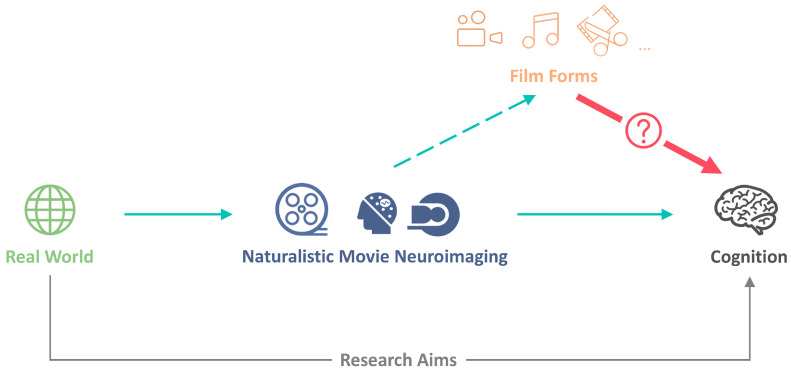
A framework for studying human cognition through film form. The research aim is to investigate human cognition in real-world contexts. Because real-world cognition is difficult to access under controlled laboratory conditions, naturalistic movie-based neuroimaging paradigms are utilized. However, movies inherently contain specific film forms, which means that observed cognitive and neural responses inevitably reflect the influence of film form. Therefore, it is necessary to systematically examine how film form shapes cognitive processing.

To accommodate this complexity, we propose a conceptual framework that distinguishes naturalistic movie neuroimaging as a research paradigm from film form as a mediating mechanism between real-world experience and cognition (Figure 1). This framework adopts a two-step perspective: movies are first analyzed in terms of the constituents of film forms, and the cognitive effects of these film forms are then examined. Within this perspective, movies are neither transparent proxies for reality nor arbitrary laboratory stimuli; they are structured stimuli whose effects on cognition are systematically shaped by film form. Accordingly, the relationship can be schematically expressed as Real World → Naturalistic Movie Neuroimaging → Film Form → Cognition, where film form organizes sensory, emotional, and narrative information before and during cognitive processing. This perspective explains the richness and interpretive difficulty of naturalistic movie neuroimaging and motivates systematic links between film form and cognition. On this basis, the following sections review empirical studies examining how specific film forms—such as editing, camera movement, and narrative structure—modulate perception, emotion, and social understanding.

### 3.2. Accumulating Evidence for Film Form as a Mediator of Cognition

Grounded in the previous framework for studying human cognition through film form, this section synthesizes empirical evidence demonstrating how film form systematically shapes cognitive processing during movie viewing (Table 1). We focus on key filmic elements, including editing, camera movement, color, and sound, which provide a useful basis for understanding their influence on cognitive domains.

(1) Editing

Accumulating evidence suggests that film editing serves as a core formal mechanism through which cinematic stimuli structure perceptual prediction, attentional allocation, and shared neural dynamics during naturalistic viewing. Rather than merely linking shots, editing reorganizes the temporal structure of perceptual input, thereby shaping how viewers anticipate, integrate, and update narrative information over time. From a predictive processing perspective, editing-induced discontinuities can be understood as formally constructed events. These constructed events generate prediction errors whose magnitude depends on narrative expectations established by the edited sequence itself, rather than on real-world continuity (Drew et al., 2023). Neural and behavioral responses at cut boundaries systematically scale with the depth and unpredictability of narrative transitions, indicating that edits actively engage predictive and conflict-monitoring mechanisms embedded within the film’s temporal design. Beyond individual prediction, editing also operates at the level of attentional coordination and collective neural dynamics. By imposing a shared temporal structure on perceptual input, edited sequences align viewers’ attentional trajectories and promote synchronized neural responses across individuals. ISC analyses demonstrate that editing enhances neural synchrony relative to unedited presentations of identical content, particularly in sensorimotor and frontal regions, suggesting that film form actively shapes population-level neural coherence during narrative engagement (Hasson et al., 2008; Herbec et al., 2015). At a finer perceptual scale, continuity editing techniques further support attentional alignment by guiding viewers’ visual expectations across cuts. Continuity is maintained not through explicit spatial reconstruction, but through attentional selection. This selection ensures pre-cut expectations remain compatible with post-cut information, allowing perceptual fluency to emerge despite physical discontinuities (T. J. Smith, 2012). Collectively, these findings indicate that editing mediates cognition by coordinating predictive processing, attentional guidance, and inter-subject alignment within a formally structured temporal context.

Beyond its effects on perceptual prediction and attention, film editing plays a central role in narrative comprehension. Event segmentation refers to the process by which continuous audiovisual input is parsed into meaningful narrative units. Evidence across behavioral and neuroimaging studies indicates that not all editing discontinuities contribute equally to segmentation. Disruptions in ongoing action exert a stronger influence on perceived event boundaries than changes in spatial or temporal context alone. This pattern suggests that continuity editing is tuned to preserve narrative coherence across spatiotemporal breaks while selectively marking meaningful transitions (Magliano & Zacks, 2011). At the neural level, event boundaries are associated with transient responses in early visual areas. More distinct engagement emerges in higher-order perceptual and associative regions when narrative structure, rather than low-level visual change, is disrupted. Event segmentation also has direct consequences for memory-related processing. During continuous movie viewing, observer-defined event boundaries robustly engage the hippocampus, with response magnitude scaling with the salience of narrative transitions and remaining reliable after controlling for perceptual confounds (Ben-Yakov & Henson, 2018). Importantly, narrative comprehension further depends on the information integration across extended temporal windows. When the temporal continuity of film narratives is disrupted, inter-subject neural synchrony decreases in regions implicated in long-timescale integration. These regions include the posterior temporal cortex, medial prefrontal cortex, precuneus, and cerebellum, highlighting their role in linking consecutive events into coherent sequences (Lahnakoski et al., 2017). These converging lines of evidence indicate that editing shapes cognitive processing not only by guiding moment-to-moment attention and prediction but also by structuring experience into discrete events and extended narrative sequences. Through its influence on event segmentation and integration, film editing functions as a key mechanism mediating narrative understanding and memory formation in naturalistic movie viewing.

Film editing exerts a powerful influence on emotional perception by embedding local cues within emotionally meaningful contexts. Rather than being read in isolation, emotions in film are inferred through contextual processing, making emotional perception a fundamentally inferential process closely aligned with social cognition. A canonical illustration of this mechanism is the Kuleshov effect, which demonstrates how viewers’ interpretations of neutral facial expressions are systematically shaped by surrounding shots (Pudovkin, 1970). Behavioral evidence shows that neutral faces are perceived as emotionally congruent with preceding or surrounding scenes in terms of valence, arousal, and categorical emotion judgments (Barratt et al., 2016; Calbi et al., 2017). High-density EEG data reveal that although faces remain neurally encoded as neutral at early perceptual stages, later components, including the Late Positive Potential, are selectively modulated by emotional context. This modulation reflects evaluative conflict or congruence between the face and the surrounding scene (Calbi et al., 2019). Extending this line of work to authentic cinematic materials, previous work provided converging behavioral and neuroimaging evidence that contextual processing via POV-based editing robustly alters emotional perceptions in naturalistic movie viewing (Cao et al., 2024b). Neural correlates of this effect spanned regions implicated in contextual processing, memory, and affective evaluation, including the precuneus, posterior cingulate cortex, hippocampus, orbitofrontal cortex, fusiform gyrus, and insula. Consistent with this contextual perspective, practical editing frameworks distinguish emotional, action-based, and rhythmic editing points, each shaping viewers’ emotional engagement across the narrative (Xing & Zhuang, 2022). Taken together, these findings support a contextual processing account of emotional perception in film editing. Editing shapes emotions not by directly inserting affective signals, but by orchestrating the relational structure through which viewers infer emotional meaning across shots and over time.

Film editing also systematically reshapes viewers’ time perception. During movie viewing, subjective time reflects perceptual sampling, attention, and narrative structure, all modulated by editing. Higher cut density accelerates information updating, leading viewers to perceive time as passing faster while overestimating its duration. This aligns with attentional and embodied timing models: rapid editing increases eye movements and shortens fixations, which are associated with overestimation of perceived duration by increasing the density of perceptual events (Balzarotti et al., 2021). Converging evidence further indicates that such editing-driven distortions of subjective time are mediated by sensorimotor timing mechanisms, with modulation of supplementary motor area activity selectively altering perceived duration, time passage, and action speed as a function of editing style (Cancer et al., 2025). Filmmakers shape subjective time through narrative-level techniques such as temporal compression, expansion, and real-time editing. Experimental evidence shows that perceived duration varies with these strategies, with expanded scenes feeling longer than real time, which feel longer than compressed, reflecting interactions between editing, visual saliency, and ongoing action rather than temporal manipulation alone (Liapi et al., 2024). These findings converge on the view that time perception in film is a higher-order cognitive construct shaped by editing-driven attentional dynamics and event-level organization.

At a level finer than the cut itself, continuity editing operates at a microstructural scale. It regulates how specific visual parameters, such as scale and camera angle, change across shot transitions. At a fundamental level, shot transitions elicit rapid neural responses even during naturalistic movie viewing. Time-resolved EEG measures reveal a characteristic sequence following cuts. An early orienting response appears as transient theta-band synchronization within the first ~200 ms. This is followed by a later phase of delta-band desynchronization associated with perceptual integration and scene updating over parietal regions (Sanz-aznar et al., 2021). Building on this temporal framework, further evidence suggests that the brain’s response to cuts is graded by the specific visual transformations introduced across shots. Within continuity editing, changes in shot scale exert a particularly strong influence on post-cut neural dynamics. ERP modulations in the N300–N400 time window indicate that scale-out cuts amplify neural responses relative to scale-preserving transitions, whereas scale-in cuts produce attenuated responses (Sanz-Aznar et al., 2023). This asymmetry mirrors subjective cut visibility: scale-in edits are more readily integrated into the ongoing scene representation, while scale-out edits generate stronger prediction errors and are more likely to be consciously detected. By contrast, comparable variations in camera angle do not elicit robust ERP differences, suggesting that angular changes are more easily accommodated within the viewer’s internal scene model. Collectively, these findings support a predictive-processing account of continuity editing at the microstructural level. From this perspective, the cognitive impact of editing lies not only in whether a cut occurs, but in how precisely its visual parameters are tuned to the brain’s expectations of continuity.

(2) Camera, lighting, and color

Stereoscopic cinematography provides a clear illustration of how camera technology alters cognition by increasing spatial inference demands. More broadly, immersive display technologies such as stereoscopic presentation and virtual reality (VR) extend this effect by embedding viewers within spatially enriched 3D environments that more closely approximate real-world perception. Neuroimaging and psychophysiological evidence converge in showing that 3D presentation amplifies activity within distributed visuospatial networks, particularly regions involved in depth computation and spatial integration. Rather than altering representational content, stereoscopic depth introduces dynamic disparity cues that must be continuously sampled and integrated, thereby increasing perceptual workload and engaging dorsal and parietal processing streams over time (A. Ogawa et al., 2013). In addition, across laboratory and immersive-environment studies, stereoscopic and high-immersion displays produce stronger physiological responses, indexed by autonomic measures and oscillatory EEG activity. However, higher-level judgments such as genre classification or narrative interpretation remain largely intact (Tian et al., 2021a; Vischsupa, 2010). Aside from perceptual enrichment, film form and stereoscopic cinematography can also affect the reviewer’s emotional experience in the VR environment. For example, unedited VR films enhance attentional stability and emotional immersion, whereas conventional editing techniques fragment attention and reduce emotional coherence (Zou et al., 2025). EEG evidence similarly shows that editing increases cognitive load and engages frontal–parietal networks associated with event segmentation, reflecting a trade-off between narrative structuring and immersive continuity (Tian et al., 2021b). At a broader scale, immersive VR experiences modulate large-scale functional brain networks, with distinct connectivity patterns supporting different emotional states (Jo et al., 2025). Moreover, emerging multimodal evidence indicates that immersive cognition cannot be fully accounted for by audiovisual factors alone. The addition of sensory channels such as olfaction enhances behavioral realism in VR, bringing responses closer to real-world behavior (Khan & Nilsson, 2023), suggesting that increasingly ecological media environments recruit broader multisensory mechanisms. Together, these findings indicate that immersive camera technologies primarily modulate a basic arousal dimension, amplifying emotional experience through enhanced perceptual load.

Beyond static depth cues, camera movement introduces a distinct embodied channel through which film form shapes cognitive processing (Gallese & Guerra, 2019). When the camera moves, it replaces viewers’ own motion signals with visually guided movement. This externally guided motion can evoke embodied simulation even in the absence of physical action, turning cinematic framing from a static compositional arrangement into a dynamic means of shaping bodily engagement. Across experimental contexts, camera movement has been shown to systematically bias spatial presence, involvement, and experiential intensity, even when narrative content is held constant. Different movement techniques, including Steadicam, dolly, and handheld motion, modulate viewers’ sense of immersion and involvement. These effects indicate that camera trajectories function as formal parameters that tune embodied engagement rather than merely conveying narrative information (Yilmaz et al., 2023). Movements that imply greater physical effort or displacement, such as ascending trajectories, further amplify perceived motion intensity, emotional involvement, aesthetic appreciation, and even subjective duration (Kolesnikov et al., 2025). At the neural level, this embodied engagement is reflected in sensorimotor dynamics rather than in attentional reorienting alone. Camera movements that simulate observer motion elicit stronger event-related desynchronization in beta-band activity over rolandic regions, a signature associated with motor resonance and bodily involvement, compared with static or purely optical transformations such as zooms (Heimann et al., 2019). Taken together, these findings suggest that moving cameras actively shape embodied experience at a pre-reflective level.

Frame rate manipulation reveals how learned perceptual expectations constrain camera-driven cognition. While higher frame rates increase objective visual smoothness, they do not automatically enhance viewer preference. Eye-tracking and behavioral data indicate that engagement depends not only on perceptual fidelity but also on internalized norms of cinematic motion. Only observers who could consciously discriminate between frame rates expressed stable preferences, which often favored the conventional 24 fps over higher rates (Pazhoohi & Kingstone, 2021). This counterintuitive finding suggests that cinematic realism is distinct from perceptual realism, as excessively smooth motion can conflict with culturally ingrained expectations of filmic movement, highlighting a tension between technological optimization and learned perceptual models.

Lighting design further shapes cognitive processing by modulating affective salience at both conscious and subliminal levels. Behavioral studies demonstrate that global lighting styles systematically bias emotional tone, with low-key lighting enhancing suspense and intensity, and high-key lighting promoting more positive affective states (Poland, 2015). At the neural level, lighting direction alone can alter early emotional processing. Variations such as underlighting, top-lighting, or silhouette configurations amplify early occipito-parietal responses associated with automatic emotional evaluation, even in the absence of explicit emotional content (Huttunen, 2025). Rather than conveying meaning directly, lighting modulates the emotional readiness with which visual information is processed.

The influence of color on emotional perception has been consistently demonstrated in psychological research. Converging evidence shows that variations in luminance and saturation systematically bias affective appraisal. Brighter and more saturated colors are generally associated with positive valence, whereas darker tones tend to elicit negative emotional responses (Suk & Irtel, 2010; Takahashi & Kawabata, 2018; Wilms & Oberfeld, 2018). In film, color therefore functions not merely as an enhancement of black-and-white imagery, but as an independent expressive dimension that actively structures emotional meaning. From this perspective, comparisons between color and black-and-white films offer a principled way to examine how chromatic information modulates emotional experience. Behavioral findings consistently indicate that color presentation enhances perceived emotional valence relative to black-and-white versions, suggesting that chromatic cues contribute affective information beyond decorative detail (Detenber et al., 2000). This modulatory role of color becomes more pronounced when considered alongside other sensory channels. When auditory input is held constant, color films, particularly in emotionally neutral contexts, reliably elicit higher valence ratings than their black-and-white counterparts (İyilikci et al., 2023). Importantly, such effects are consistent with evidence that color is bound as an integral component of visual representations at early stages of processing, thereby strengthening perceptual encoding independently of higher-level semantic interpretation (Spence et al., 2006). Extending beyond color in isolation, recent evidence further indicates that emotional perception in film reflects interactions between visual form and temporal structure, with color and editing jointly modulating affective responses at both behavioral and neural levels (Cao et al., 2024a). In summary, these results suggest that film color shapes emotional perception primarily through low- to mid-level perceptual mechanisms, with affective modulation emerging from variations in saturation in color films and from gradations of luminance in black-and-white imagery.

(3) Sound, subtitle, narrative, and viewer-film interaction

One fundamental contribution of sound lies in its capacity to construct spatial presence and guide perceptual continuity without relying on visual immersion alone. Spatialized audio extends the perceived environment beyond the visual frame by recruiting sensorimotor and embodied processing systems, enhancing the subjective feeling of “being there” even when viewers remain physically stationary. Neurophysiological evidence shows that surround sound amplifies neural signatures of bodily engagement and action readiness in centro-parietal regions, indicating that auditory space functions as a mediating scaffold that stabilizes embodied presence and perceptual continuity across cuts and camera changes (Langiulli et al., 2023). Beyond spatial presence, sound and music play central roles in shaping interpretive and emotional alignment over time. Rather than eliciting isolated affective reactions, musical soundtracks systematically bias how viewers construe characters, environments, and narrative trajectories by modulating vigilance, emotional framing, attentional focus, and anticipatory judgments (Ansani et al., 2020). At the neural level, moment-to-moment acoustic features of film music reliably track shared fluctuations in arousal and valence across viewers, with convergent activity in integrative hubs such as the superior temporal sulcus and the precuneus, which bind musical dynamics to evolving narrative context and support inter-subjective emotional coherence (Muller-rodriguez & Daly, 2021). Sound further contributes to narrative comprehension by scaffolding predictive inference. Auditory cues embedded in film soundtracks signal causal structure and impending events, reducing uncertainty when visual information is ambiguous or temporally extended. When such cues are removed, predictive accuracy declines and becomes more dependent on individual cognitive resources, underscoring sound’s role in stabilizing narrative expectations rather than conveying explicit content (Hutson et al., 2021). Crucially, auditory information also interacts with visual continuity mechanisms at a pre-attentive level. The presence of sound substantially reduces awareness of edits by sustaining attentional continuity across cuts, effectively masking visual discontinuities and maintaining perceptual fluency, particularly when audio aligns with post-cut motion (T. J. Smith & Martin-Portugues Santacreu, 2017). Together, these findings converge on the view that sound functions as a multimodal organizing force that stabilizes spatial presence, aligns emotional interpretation, supports predictive inference, and sustains perceptual continuity, coordinating embodied, affective, and interpretive processes during naturalistic movie viewing.

While sound primarily modulates affective and embodied dimensions, subtitles introduce a linguistic layer that directly competes for visual attention and restructures multimodal integration. Eye-tracking evidence indicates that subtitle reading is highly automatic and difficult to suppress, regardless of viewers’ language proficiency (Bisson et al., 2014). As a result, subtitles systematically reallocate attentional resources within the visual field, imposing a stable reading rhythm that interacts with cinematic composition and editing. Crucially, subtitles do not merely distract from the image. By foregrounding linguistic information, they reshape narrative construction, altering the relative salience of visual elements and redistributing interpretive weight across competing narrative cues (Kruger, 2012).

At a higher level, understanding a film’s narrative provides a structure that organizes embodied, emotional, and social–cognitive processing over time. As viewers piece together the story, even in films without explicit exposition, their sensorimotor systems become increasingly involved, indicating embodied simulation of characters’ actions and intentions (Y. Ogawa & Shimada, 2016). Activity in premotor and supplementary motor areas scales with narrative comprehension and individual empathy, suggesting that empathic engagement emerges from accumulated narrative meaning rather than from isolated emotional cues. Narrative coherence also supports higher-order social cognition by guiding how viewers infer characters’ mental states (Cabañas et al., 2023). When the story creates differences in knowledge between the audience and characters, viewers spontaneously make theory-of-mind inferences, integrating others’ beliefs and knowledge into their evolving understanding of events. This is especially true when prior context allows spectators to anticipate characters’ false beliefs. Such findings show that social–cognitive engagement depends on the narrative as a whole rather than on moment-to-moment perceptual cues. In this way, narrative does more than convey a story. It actively shapes the mental space in which viewers simulate intentions, beliefs, and motivations. At the population level, narrative coherence promotes shared meaning by aligning neural activity across viewers (Somech et al., 2022). Naturalistic films elicit synchronized responses in prefrontal regions involved in interpretation, evaluation, and affective appraisal, tracking collective experiences such as humor independently of low-level sensory features. Overall, these findings show that narrative acts as a slow, integrative constraint, structuring embodied simulation, spontaneous social inference, and shared neural alignment during movie viewing.

The viewer-film interaction, characteristic of immersive cinema, extends traditional audiovisual approaches beyond the reliance on sensory channels, constituting a distinct film form. This interactive dimension makes film viewing more closely approximate the naturalistic cognition. Interaction can take multiple forms, broadly categorized into dialogic structure within the film (e.g., whether characters address each other or directly address the viewer), explicit viewer input, and real-time social engagement. First, dialogue carries dynamic social and emotional information that unfolds over time, including whether communication is directed between characters or toward the viewer, which differentially affect the viewer’s social engagement. Emotional alignment and divergence between speakers evolve systematically across narratives, depending on how viewers track interpersonal dynamics and social meaning (Hipson & Mohammad, 2021). Experimental work further suggests that embodied interaction contexts, such as interactive movie environments, modulate the sense of presence (Jun Hu, 2008). Second, by requiring viewer contingent reaction to the film, interaction introduces an active decision layer that engages cognitive and social evaluation processes. Behavioral paradigms using simple film stimuli show that participants can continuously report perceived social interaction through button responses, revealing that such judgments depend on both low-level motion cues and higher-order social understanding (Rasmussen & Jiang, 2019). In more complex interactive environments, such as video games, real-time input engages more distributed brain networks associated with action monitoring, conflict processing, and affective regulation, reflecting continuous brain–environment coupling compared with passive stimulus processing (Mathiak & Weber, 2006). Finally, real-time social interaction can further engage interpersonal neural alignment. Hyperscanning and live-interaction studies show that prior conversation and co-presence increase inter-brain synchrony during subsequent co-viewing, particularly in regions associated with social cognition such as the temporoparietal junction (TPJ)(De et al., 2024). Direct face-to-face interaction recruits social and reward-related networks, including the TPJ, superior temporal sulcus, anterior cingulate cortex, and ventral striatum, emphasizing the importance of contingent responding and joint attention in naturalistic social cognition (Redcay et al., 2010), thereby bringing experimental paradigms closer to real-world social interaction. Together, these findings suggest that film cognition frameworks should incorporate interactive dimensions towards better ecological validity.

### 3.3. Construct the Film Cognition Matrix

Building on the previous framework for studying human cognition through film form, accumulated evidence demonstrates that film form exerts systematic and domain-specific influences on cognition during naturalistic viewing. However, existing research remains conceptually fragmented (Table 1). This limitation stems not from a lack of empirical findings, but from the absence of a principled framework for organizing how different film forms relate to distinct cognitive functions in a cumulative manner. Most studies focus on isolated variables within narrowly defined cognitive contexts, which limits our ability to understand how multiple film forms jointly shape cognitive processing over time. Film form is rarely conceptualized as a structured system operating across cognitive domains. From a neural perspective, this fragmentation is reflected in limited mechanistic accounts: brain-based evidence often remains descriptive, and experimental designs are insufficiently sensitive to dynamic coordination across cognitive systems. Film forms are typically treated as static stimulus features rather than as interacting components that jointly modulate attention, emotion, and memory. As a result, current research lacks an integrative account of how film form maps onto cognitive and neural architecture, even though converging evidence indicates interaction among these dimensions during naturalistic viewing.

**Figure 2:**
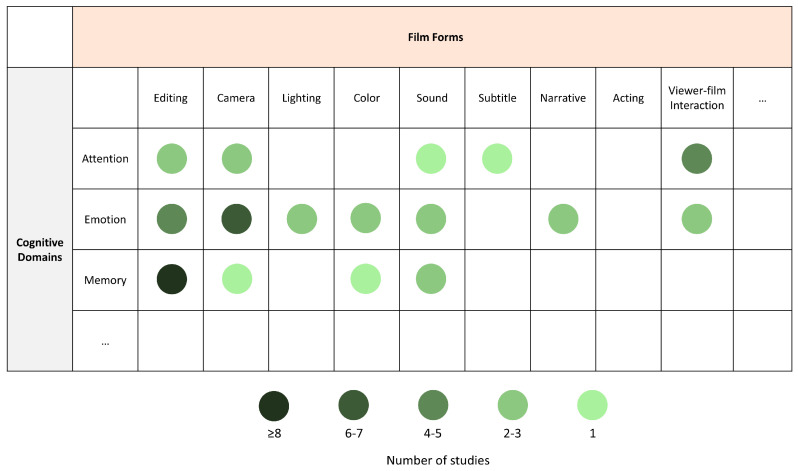
Illustration of the Film Cognition Matrix. The matrix is organized along two orthogonal dimensions: Film Forms, which encompass core expressive components of cinematic language, such as editing, camera, lighting, color, sound, subtitles, narrative, acting and viewer–film interaction; and Cognitive Domains, such as attention, emotion, and memory. The green circles within the matrix represent domains that have been empirically studied, with darker colors indicating a greater number of existing studies. Blank areas indicate domains that remain underexplored and require further investigation.

To address this gap, we propose the Film Cognition Matrix, as shown in Figure 2. Rather than cataloging film forms, the matrix defines an analytic space that maps core film forms onto core cognitive domains engaged during naturalistic viewing. The matrix is organized along two orthogonal axes: film forms (e.g., editing, camera, lighting, color, sound, subtitles, narrative, acting, and viewer-film interaction) and cognitive domains (e.g., attention, emotion, memory). Each cell represents a theoretically motivated form–function intersection, formalizing the assumption that specific film forms selectively modulate specific cognitive domains (Figure 1). The matrix extends existing process-oriented models of film comprehension, such as Scene Perception and Event Comprehension Theory (SPECT) (Loschky et al., 2018, 2020), by specifying which visual stimuli are likely to drive which cognitive mechanisms. While SPECT characterizes the temporal dynamics of movie narrative processing, the matrix focuses on form–function correspondence, making SPECT and the matrix complementary. As a conceptual scaffold, the matrix allows existing findings to be repositioned as systematic intersections of film form and cognition. Examples include research on editing and event segmentation (Magliano & Zacks, 2011; Zacks et al., 2010), as well as studies linking editing, color, and emotional perception (Cao et al., 2024a), which can be understood as structured film-form–cognition mapping rather than isolated stimulus effects. At the same time, it highlights critical gaps in the literature, particularly the scarcity of studies that systematically compare multiple film forms within the same cognitive domain or examine how different domains interact dynamically over time. Methodologically, the matrix motivates experimental designs that combine continuous movie viewing with targeted manipulations of film forms, thereby enabling more mechanistic investigations of how film forms mediate shared cognitive and neural responses under ecologically valid conditions.

## 4. Discussion

### 4.1. Editing as the Dominant Film Form Shaping Cognitive Processing

Although multiple film forms contribute to cognitive processing, the reviewed literature reveals a clear methodological and theoretical asymmetry in research focus, with studies examining editing substantially outnumbering those addressing other film forms such as color, camera, or sound (Table 1). This asymmetry reflects not only research tradition but also the distinctive theoretical role of editing within film forms. First, editing occupies a central position in film language by defining the temporal architecture of cinematic experience (Bordwell & Thompson, 2008). Rather than merely linking shots, editing actively segments, reorganizes, and recomposes perceptual input over time. From this perspective, empirical work converges on the view that editing-induced discontinuities function as formally constructed events that shape predictive processing during movie viewing (Drew et al., 2023). Cuts generate prediction errors whose magnitude depends on narrative expectations established by the edited sequence itself, rather than on real-world continuity. Neural and behavioral responses at cut boundaries systematically scale with the depth and unpredictability of narrative transitions, implicating predictive and conflict-monitoring mechanisms embedded in the film’s temporal design. Second, editing also marks a fundamental divergence between filmic experience and everyday perception (Andreu-Sánchez et al., 2017, 2024). Whereas real-world cognition unfolds within continuous spatiotemporal environments, cinematic experience is inherently fragmented and recomposed through cuts, transitions, and montage (Herbec et al., 2015; Jääskeläinen et al., 2021), introducing externally imposed event boundaries that do not arise from natural environmental structure. Neuroimaging evidence shows that such cut boundaries, consistent with broader event segmentation mechanisms, reliably elicit hippocampal responses predictive of subsequent memory encoding (Ben-Yakov & Henson, 2018), as well as deactivation of posterior temporal lobes activity associated with narrative updating (Lahnakoski et al., 2017). Taken together, these findings position editing as a privileged site for examining how artificial temporal segmentation reshapes core cognitive processes, including memory organization and narrative comprehension. Third, this theoretical centrality is further reinforced by methodological considerations, editing operations naturally afford discrete, time-lockable events, allow systematic manipulation of identical visual content, and entail relatively low stimulus-construction cost, making them especially compatible with event-related ERP and fMRI designs (Magliano & Zacks, 2011; Sanz-Aznar et al., 2023). Finally, the existence of films that minimize or suppress editing, such as long-take and continuous-shot films (e.g., 1917, Birdman), highlights unresolved questions concerning how cognitive segmentation emerges from non-cut-based cues despite preserved visual continuity (Ghosh, 2022). In such cases, viewers still construct event structure, emotional dynamics, and narrative coherence. The neural mechanisms supporting segmentation under these conditions remain poorly understood and represent a promising direction for future research.

### 4.2. Approaching Reality in Movie Neuroimaging: Cinema as an Asymptotic Approximation to Reality

Conceptually, naturalistic is defined as that which represents what is real. One cannot set a hard boundary between naturalistic and artificial stimuli. Instead, naturalistic stimuli are more appropriately conceptualized along a gradient towards the true naturalistic. Therefore, the relationship between film and naturalistic paradigms becomes central: Are films themselves naturalistic, or do they merely approximate natural experience? Drawing on film theorist André Bazin’s ontological account of cinema (Bazin, 2005), which characterizes film as grounded in indexical relations to reality rather than as mere representation, cinema can be understood as an asymptotic approximation to the real—an ever-approaching yet non-identical form. This perspective has important implications for naturalistic neuroimaging, where films are often treated as proxies for real-world experience. Empirical evidence, however, suggests that such an approximation is partial and systematically constrained. For example, reduced blink rates and weaker Mu-band desynchronization during screen viewing indicate diminished embodied engagement compared to real-world contexts (Andreu-Sánchez et al., 2017, 2024; Martín-Pascual et al., 2018). Accordingly, the critical question is not whether films are naturalistic, but where they lie along a continuum of approximation and under what conditions they approach real-world cognition. This reframing shifts the focus from categorical distinctions between natural and artificial stimuli to the balance between ecological validity and experimental control. While simplified laboratory stimuli isolate specific mechanisms, films preserve key structural properties of real-world experience, including temporal continuity, multisensory integration, and social interaction (Cao et al., 2026; Hasson et al., 2008). Within this asymptotic framework, we argue that film form functions as a mediating mechanism that structures information flow and constrains cognitive processing (Figure 1) and that mapping specific film forms onto cognitive domains can provide a systematic account of how films approximate real-world cognition (Figure 2). Specifically, early research on human cognition predominantly relied on static images as experimental stimuli. However, due to their limited ecological validity and their relative distance from real-world experience, dynamic film stimuli were subsequently introduced as a more naturalistic form of visual representation. Films capture spatiotemporal information through camera movement and organize it through editing, thereby enabling viewers to become immersed in a simulated environment within which cognitive processes can be studied more naturally (Herbec et al., 2015; Kolesnikov et al., 2025). This transition marked a significant improvement in ecological validity. Nevertheless, film viewing remains constrained by a fixed frame and predetermined viewpoint, which limit viewers’ ability to freely explore the environment and thereby reduce perceptual autonomy relative to real-world experience. To address this limitation, VR was introduced. Immersive VR environments couple perception and action, allowing users to actively explore their surroundings and thus enabling more ecologically grounded yet experimentally controlled investigations of cognition (Bohil et al., 2011; Thurley, 2022). Beyond perceptual immersion, real-world cognition fundamentally involves continuous interaction with the environment. To better approximate this aspect, interactive films have emerged, enabling users to influence narrative progression and environmental dynamics. This progression reflects a shift from observation-based to participation-based paradigms, in which neural activity increasingly reflects brain–environment coupling rather than responses to externally imposed stimuli (Burelli & Dixen, 2024; Ninaus et al., 2014; Wolfe et al., 2022). From this perspective, the objective of naturalistic movie neuroimaging is not to eliminate the gap between film and reality, but to characterize how this gap is structured and how different film forms position stimuli along an asymptotic trajectory toward real-world cognition.

### 4.3. Leveraging Film-Form–Cognition Mapping for Stimulus Selection and Experimental Control

Film-form–cognition mapping is inherently bidirectional: film form not only informs neural interpretation but can also be systematically manipulated as experimental variables to engage specific cognitive processes. Accordingly, this perspective reframes film form from a potential confound into a controllable, theory-relevant dimension of experimental design.

From the perspective of stimulus selection, film form must be carefully evaluated when examining specific cognitive processes. Researchers may minimize confounding features or use condition-based contrasts to isolate their effects. Although such control is not always central to naturalistic paradigms, it becomes essential for fine-grained cognitive specificity. Film-form–cognition mapping thus provides a principled basis for selecting or comparing stimuli based on expected cognitive engagement. For example, camera movement influences embodied simulation and attention (Heimann et al., 2019; Kolesnikov et al., 2025), whereas color modulates affective arousal (İyilikci et al., 2023). With these mapping, research can systematically guide stimulus selection, design-controlled contrasts between filmic features, and more precisely link formal manipulations to targeted cognitive processes.

Beyond selection, this logic extends to the construction of purpose-built stimuli aligned with experimental hypotheses. In a child–parent fMRI study, Su et al. (2024) used a 6 min conflict scenario depicting an argument between a 7-year-old girl and her mother. The film was deliberately produced with controlled framing and shot composition to align socioemotional content with experimental objectives, and its storyboard structured segmentation into discrete narrative events, aligning analytic units with cinematic boundaries. Analysis of intersubject neural synchrony showed that negative family emotional climate predicted reduced child–parent vmPFC–hippocampal coupling during viewing, illustrating how film form informs both stimulus design and analysis. More broadly, this approach supports a shift from post hoc interpretation to prospective control through systematic manipulation of filmic variables. For instance, filming perspective differentiates first-person experienced emotion from third-person portrayed emotion, engaging distinct cognitive and neural processes (Baveye et al., 2015; Labs et al., 2015; Morgenroth et al., 2023; Uhrig et al., 2016). Our ongoing collaboration with a psychiatric hospital similarly employs purpose-built point-of-view paradigms (unpublished data), aligning stimulus structure with socioemotional hypotheses. In addition to live-action filming, emerging AI video generative tools (e.g., Seedance 2.0, https://seedance.io/seedance-2, accessed on 16 February 2026) enable rapid production of storyboard-synchronized, parametrically controllable stimuli. These tools allow targeted manipulation of filmic parameters (e.g., pacing, perspective, audiovisual alignment) while preserving dynamic, multimodal structure. Rather than reverting to simplified laboratory paradigms, such approaches establish a continuum between naturalistic and controlled designs, enabling isolation of causal mechanisms without sacrificing ecological validity. Taken together, these advances further position film-form–cognition mapping as a practical framework for both stimulus construction and experimental control.

### 4.4. Modeling Film-Form–Cognition Mapping in Neuroimaging Data Analysis

Firstly, we should clarify that the ultimate goal is to understand naturalistic cognition as an asymptotic target, toward which film-based paradigms progressively approximate real-world experience. In this review, we position film-form–cognition mapping as a central methodological and theoretical contribution to this goal. Within this objective, modeling film-form–cognition mapping provides a principled means to refine interpretation and enable comparisons across levels of naturalism. Neural responses during movie viewing reflect not only real-world cognitive processes but also those induced by film form, requiring careful interpretation (Jääskeläinen et al., 2021; Sonkusare et al., 2019). Rather than treating film forms as distortions, we conceptualize film form as a structured pathway through which reality is selectively organized and rendered experimentally accessible (Figure 1). Films thus provide a controlled yet ecologically rich environment in which perceptual, affective, and social–cognitive processes unfold over time, preserving key dynamic properties of natural experience (Bordwell et al., 1993; Tan, 2018). Accordingly, neural activity during movie viewing reflects real-world cognition as structured and temporally coordinated by film form.

Within this framework, the key analytical step is to explicitly model filmic features to account for factors shaping neural responses. Operationally, this involves three steps: annotating film forms, encoding them as time-resolved predictors, and incorporating them into neuroimaging models. For example, editing can be modeled by coding shot boundaries as discrete events and deriving continuous measures such as shot duration or editing rate, which can be convolved with a hemodynamic response function in fMRI analyses. Camera movement can be quantified using motion energy or optical flow algorithms to generate frame-by-frame estimates of visual motion intensity, while shot scale can be annotated either categorically (e.g., close-up, medium shot, long shot) or continuously based on the relative size of faces or objects within the frame. Similarly, color properties can be operationalized through low-level features such as luminance, saturation, and hue distributions extracted computationally across frames. These features can then be modeled as event-based or continuous regressors, allowing their contributions to neural activity to be statistically estimated, controlled, or compared, thereby isolating the influence of film form on brain responses.

Two complementary modeling strategies can be applied. The first is a within-film approach, in which filmic features are densely annotated within a single movie and treated as covariates. This approach enables the examination of how moment-to-moment variations in film form dynamically shape neural activity while preserving narrative continuity. For instance, fluctuations in motion energy and shot duration can be modeled alongside neural time series to test whether activity in visual or parietal regions tracks camera movement independent of narrative content, while regressors indexing the occurrence of close-up shots can be used to examine modulation in face-processing or affective networks. The second is an across-films approach, in which stimuli with similar narrative content but systematically varying film form are compared. This allows for difference-based inference that attributes neural variation to film form rather than content. For example, two versions of the same narrative can be constructed with identical content but different editing rhythms (e.g., fast versus slow cuts) or camera perspectives (e.g., first-person versus third-person), enabling direct comparison of neural responses to isolate the effects of film form. This approach can also be extended across multiple datasets, supporting scalable and reproducible comparisons of film-form effects.

Together, these strategies implement subtractive control within films and differential inference across films, whose integration is necessary to balance ecological validity and experimental control. This framework reframes neural activity during movie viewing as cognition embedded in a structured audiovisual environment (Andreu-Sánchez et al., 2024; Herbec et al., 2015). Modeling enables partial isolation of film-form effects, allowing researchers to control or directly compare them. If neural activity covaries with specific film features, this indicates the modulation of underlying cognitive mechanisms rather than the engagement of distinct forms of cognition. In summary, modeling film-form–cognition mapping improves the analytical precision of naturalistic movie neuroimaging, thereby aligning with the asymptotic goal of characterizing and reducing the gap between film and reality.

### 4.5. From Film-Form–Cognition Mapping to Neurocinematics and Neuroaesthetics

Building on film-form–cognition mapping, this review extends its implications to neurocinematics by positioning film form as a central mechanism through which complex audiovisual stimuli shape cognitive processing under naturalistic conditions. Current review focuses on how film form systematically shapes cognitive processing, aligning with the core aims of neurocinematics, which examines how the brain processes complex audiovisual media under naturalistic conditions. Since the foundational work of Hasson and colleagues (Hasson et al., 2008), neurocinematic studies have demonstrated that films robustly synchronize neural activity across viewers, revealing shared patterns of attention, engagement, and narrative processing at the population level (Hasson et al., 2010b; Jääskeläinen et al., 2021; Lahnakoski et al., 2014; Redcay & Moraczewski, 2020). Recent editorials have further framed neurocinematics as an interdisciplinary effort to understand continuous audiovisual processing in ecologically valid contexts, with implications for media creation, experimental design, and clinical applications (Andreu-Sánchez et al., 2023, 2025). However, despite these advances, existing overviews largely emphasize neural alignment while offering limited theoretical integration of how the internal structure of film stimuli organizes cognition across domains. Although Andreu-Sánchez provides a comprehensive synthesis of recent findings, a principled framework linking film properties to cognitive mechanisms remains underdeveloped. Building on this literature, the present review systematizes empirical findings across film forms and cognitive domains (Table 1) and proposes the Film Cognition Matrix to map film forms onto cognitive processes in a many-to-many manner (Figure 2). This synthesis highlights that cognitive processing during movie viewing emerges from interactions among multiple film forms rather than from isolated features, jointly shaping attention, emotion, and memory over time. Importantly, this framework addresses a key methodological limitation in current naturalistic movie neuroimaging research: while films are often analyzed in terms of individual formal variables, audiovisual information is processed as an integrated stream, causing interactions among editing, camera movement, sound, and color to be absorbed into holistic neural responses and remain insufficiently characterized (Tan, 2018). Recent studies increasingly underscore the nonlinear interaction of film forms during naturalistic viewing (Cao et al., 2024a), pointing to a central challenge in the field. By treating film form as an explicit mediating layer between audiovisual input and cognitive interpretation, the Film Cognition Matrix provides a structured foundation for integrating empirical findings and advancing mechanistic explanations of film–brain relationships.

Extending beyond neurocinematics, film-form–cognition mapping also provides a foundation for understanding neuroaesthetic processes, in which film form itself becomes an independent object of inquiry into how aesthetic experience is constructed and communicated. Extending this logic, as we treat film form as a mediating mechanism for approximating real-world cognition, it becomes necessary to consider another aesthetic mechanism. Movie viewing affords a multidimensional experiential space that integrates perception, emotion, prediction, social cognition, and narrative self-processing over extended timescales, rather than reproducing natural perception in a one-to-one correspondence (Meer et al., 2020). This view aligns with neuroaesthetic accounts emphasizing that aesthetic experience emerges from the interaction among sensory–motor processing, affective valuation, and meaning-based interpretation, rather than from any single neural system (Chatterjee & Vartanian, 2014). From this perspective, different films and genres can be understood as instantiating distinct neuroaesthetic regimes, defined by characteristic patterns of temporal integration, affective modulation, and cognitive demand imposed through formal design rather than solely by narrative themes. Comedy provides an illustrative example, as comedic experience depends on the orchestration of expectation, violation, and resolution across extended temporal windows, placing sustained demands on integrative and predictive processing. Consistent with this account, humor appreciation engages prefrontal and frontal pole regions associated with long temporal receptive windows (Jääskeläinen et al., 2016), and naturalistic studies show that humorousness covaries with prefrontal activity beyond low-level sensory properties (Somech et al., 2022). At a broader scale, full-length romantic comedy–drama films recruit distributed networks related to embodied self-processing, narrative identity, and visual attention, with individual differences in well-being reflected in distinct patterns of engagement (Ma & Skipper, 2025). These findings indicate that film form and genre systematically organize aesthetic experience by shaping the temporal unfolding of cognitive and affective processes. In addition, emerging neuroaesthetic research further supports this view by highlighting the role of reward-related and aesthetic evaluation networks, including orbitofrontal and default-mode regions, in mediating aesthetic appraisal across modalities (Chatterjee & Vartanian, 2014; Z. Chen et al., 2021), thereby positioning neuroaesthetic effects as systematic mechanisms through which films structure perception, emotion, and meaning. From an applied perspective, this framework also suggests that film form can be systematically leveraged in the film industry to optimize communication efficiency, emotional impact, and audience engagement, thereby linking neuroaesthetic mechanisms to practical questions of cinematic expression and storytelling effectiveness.

## 5. Conclusions and Future Directions

In this review, we propose a conceptual framework for naturalistic movie neuroimaging in which film form functions as a mediating mechanism between real-world experience and cognition. Based on this framework, we systematically review empirical studies examining the effects of film form on different cognitive domains and organize them into the Film Cognition Matrix. Building on this framework and the matrix, studies using naturalistic movie neuroimaging can more clearly interpret neural responses by considering the effects of film form and by systematically manipulating film stimuli to more selectively engage specific cognitive processes.

Several future directions could be drawn from the basic conceptual framework proposed in this review. First, at the level of interpretation, an important concern is how to model the influence of film form on cognition in naturalistic movie neuroimaging data. As clarified by the present framework, neural activations observed during movie viewing may reflect both processing driven by specific formal properties of the film stimulus and the target cognitive function itself (Andreu-Sánchez et al., 2017; Sonkusare et al., 2019). Despite this insight, current analytic approaches often remain focused on correlating neural activity with putative cognitive constructs, leaving a persistent explanatory gap between observed responses during movie viewing and the mechanisms underlying real-world cognition (Andreu-Sánchez et al., 2024). This gap arises because film form is rarely treated as an explicit mediating variable in data interpretation. Addressing this challenge, therefore, requires methodological advances that explicitly model and attenuate form-driven neural effects. Computational and AI-based approaches offer promising tools for modeling and partially isolating the contribution of film form to neural activity (Fu et al., 2025; Schmälzle & Huskey, 2023), thereby enabling a clearer dissociation between form-related processing and cognition-related signals in naturalistic neuroimaging analyses.

Second, addressing this interpretive challenge also requires a more systematic empirical mapping of film form-to-cognitive domains. Although existing studies have filled certain cells of the Film Cognition Matrix (Figure 2), substantial gaps remain. Many relationships between specific film forms and cognitive domains, such as the effects of acting style on attentional allocation, have yet to be systematically investigated. Extending empirical research to these underrepresented film-form–cognition mapping is essential for enriching the matrix and establishing a more comprehensive account of how film form shapes cognitive processing during naturalistic viewing. Progress in this direction depends critically on advances in multimodal measurement and cross-level integration, as cognitive activity during movie viewing unfolds across multiple temporal scales and physiological levels. Future research should therefore integrate multimodal measures—including fMRI, EEG, electrodermal activity, eye-tracking, and facial expression analysis—within unified experimental designs grounded in shared hypotheses about film form and cognition (Hu & Mohsenzadeh, 2025; Huizeling et al., 2023; Resonance et al., 2020; Tian et al., 2021a). When aligned within the matrix framework, multimodal data can provide converging evidence for the same theoretical cells, enabling examination of whether film form exerts convergent or dissociable influences across cognitive processes. The cumulative research value of the Film Cognition Matrix depends on shared data resources and standardized stimuli, as large-scale, openly available datasets provide the foundation for cross-study comparison and replication (Gong et al., 2023; Jung et al., 2025; Lahner et al., 2024; Telesford et al., 2023). Furthermore, the refinement of the Film Cognition Matrix also necessarily involves engagement with the ontology of film and with film researchers themselves. Film art is inherently open and interdisciplinary (Bordwell et al., 1993; Tan, 2018); within this broader context, film scholars and creators can also draw upon the Film Cognition Matrix to better understand viewer cognition and incorporate audience-informed feedback into creative practice (Cao et al., 2024a; Hasson et al., 2008). Such reciprocal feedback facilitates the integration of art and science, while simultaneously providing a broader foundation for the more precise manipulation of film-form variables in service of cognitive research goals. Moreover, it lays important groundwork for the development of curated film libraries with multi-layered annotation specifically designed for naturalistic movie neuroimaging.

Third, naturalistic paradigms are increasingly extending toward interactive and fully immersive environments, as reflected in the rise in interactive and game films. Although naturalistic movie neuroimaging enhances ecological validity relative to traditional laboratory tasks, it remains limited in capturing core features of real-world cognition, including contingent interaction, multisensory integration, and active participation. Accordingly, recent progress has extended film-based paradigms toward interactive formats. Interactive films and game films exemplify this shift by allowing audiences to influence narrative progression through active choices (Figueiredo & Sousa, 2023; Zhu & Zhang, 2023). VR further advances this trajectory by establishing a closed-loop system in which perception and action are dynamically coupled. Unlike passive film viewing, VR affords real-time interaction, allowing concurrent manipulation of sensory input and behavioral output, thereby approximating real-world cognition while preserving experimental control (Bohil et al., 2011; Cao et al., 2017; Thurley, 2022). In addition, VR supports multimodal integration and large-scale spatial environments, enabling controlled investigation of embodied and socially situated cognition (Lenormand & Piolino, 2022; Peng et al., 2024; Thomas et al., 2017). This progression from film to interactive media and ultimately to VR reflects a shift from observation-based to participation-based paradigms. Whereas film form organizes perception through predefined audiovisual structures, VR introduces contingent and adaptive dynamics through which cognitive processing unfolds via continuous interaction between the individual and the environment. Consequently, neural activity in VR increasingly reflects brain–environment coupling rather than responses to externally imposed stimuli. Extending this framework, research on video games and interactive digital narratives further demonstrates how narrative experience can be shaped by user input. Game-based environments elicit sustained engagement, real-time decision making, and adaptive feedback loops, all of which are closely linked to distributed neural activity patterns (Burelli & Dixen, 2024; Ninaus et al., 2014). Similarly, interactive storytelling in VR allows narrative trajectories to evolve based on user actions, amplifying emotional engagement and self-referential processing beyond those observed in linear film narratives (Wolfe et al., 2022). In summary, these developments move film-based paradigms beyond passive observation toward interactive, ecologically grounded models of cognition, narrowing the gap between controlled experimentation and real-world experience.

A fourth direction that requires further clarification concerns the conceptual boundaries articulated in the framework for studying human cognition through film form (Figure 1), particularly the tension between “returning to reality” and “returning to aesthetics.” This issue follows directly from the preceding interpretive and empirical considerations (Bordwell et al., 1993; Chatterjee & Vartanian, 2014), as a fundamental challenge of naturalistic film paradigms lies in the dual status of film as both a proxy for real-world cognition and a highly aestheticized, formally constructed object. This tension is conceptual rather than merely methodological, because it directly concerns what film cognition research can legitimately claim to explain. Hybrid experimental designs provide a practical way to address this tension by enabling causal tests of film form within intact narrative contexts (Cao et al., 2024a; Herbec et al., 2015; Lahnakoski et al., 2012). By parametrically manipulating specific aspects of film form while holding semantic and narrative content constant, these designs move beyond treating films as indivisible stimuli and make film-form–cognition mapping experimentally testable. For example, the same social interaction scene can be presented either with a continuous, observer-like camera perspective or with systematically inserted shot–reverse shot structures that exaggerate point-of-view alignment, while preserving identical dialogue, actions, and outcomes. If neural and cognitive responses remain stable across versions, this would support a return-to-reality interpretation, suggesting that viewers primarily process the situation itself. Conversely, systematic differences in neural synchronization, mentalizing-related activity, or affective engagement would indicate a return-to-aesthetics effect, reflecting sensitivity to cinematic construction rather than situational meaning. Importantly, such manipulations preserve the experiential integrity of cinematic viewing, while allowing researchers to distinguish effects that generalize to real-world situation processing from those that arise from film’s formal construction. In this sense, the framework not only organizes existing findings but also guides experimental design by clarifying the boundary between film as a proxy for reality and film as an aesthetic system.

## Figures and Tables

**Table 1:** Accumulating evidence for film form as a mediator of cognition.

Studies	Film Forms	Cognitive Domains	Effects
(T. J. Smith, 2012)	editing	attention	+
(Herbec et al., 2015)	editing	attention/editing perception	+
(Magliano & Zacks, 2011)	editing	memory/event segmentation	+
(Lahnakoski et al., 2017)	editing	memory/event segmentation	+
(Ben-Yakov & Henson, 2018)	editing	memory/event segmentation	+
(Drew et al., 2023)	editing	memory/content prediction	+
(Balzarotti et al., 2021)	editing	memory/time perception	+
(Liapi et al., 2024)	editing	memory/time perception	+
(Cancer et al., 2025)	editing	memory/time perception	+
(Sanz-aznar et al., 2021)	editing	memory/shot changes	+
(Sanz-Aznar et al., 2023)	editing	memory/shot changes	+
(Barratt et al., 2016)	editing	emotional perception	+
(Calbi et al., 2017)	editing	emotional perception	+
(Calbi et al., 2019)	editing	emotional perception	+
(Cao et al., 2024b)	editing	emotional perception	+
(A. Ogawa et al., 2013)	3D cinematography	audiovisual perception	+
(Vischsupa, 2010)	3D viewing	emotion	+
(Tian et al., 2021a)	3D viewing	emotion	+
(Tian et al., 2021b)	3D viewing	memory/event segmentation	+
(Zou et al., 2025)	3D viewing	emotion	+
(Jo et al., 2025)	3D viewing	emotion	+
(Heimann et al., 2019)	camera movement	attention	+
(Yilmaz et al., 2023)	camera movement	emotional response	+
(Kolesnikov et al., 2025)	camera movement	emotional engagement	+
(Pazhoohi & Kingstone, 2021)	frame rate	attention	-
(Poland, 2015)	lighting	emotion	N/A
(Huttunen, 2025)	lighting	emotion	+
(Detenber et al., 2000)	color	emotion	+
(İyilikci et al., 2023)	color	emotion	+
(Spence et al., 2006)	color	memory	+
(Cao et al., 2024a)	color and editing	emotional perception	+
(Hutson et al., 2021)	sound	memory	+
(Langiulli et al., 2023)	sound	memory/sound type	+
(Ansani et al., 2020)	sound	emotion	+
(T. J. Smith & Martin-Portugues Santacreu, 2017)	sound	attention/editing perception	+
(Muller-rodriguez & Daly, 2021)	music	emotional perception	+
(Bisson et al., 2014)	subtitle	attention	+
(Kruger, 2012)	audiovisual translation	attention	+
(Y. Ogawa & Shimada, 2016)	narrative	emotion/empathy	+
(Somech et al., 2022)	narrative	emotion/humorousness	+
(Cabañas et al., 2023)	narrative	emotion/irony	+
(Mathiak & Weber, 2006)	viewer–film interaction	attention	+
(Jun Hu, 2008)	viewer–film interaction	attention/presence	+
(Rasmussen & Jiang, 2019)	viewer–film interaction	attention	+
(Redcay & Moraczewski, 2020)	viewer–film interaction	attention	+
(Hipson & Mohammad, 2021)	viewer–film interaction	emotion	+
(De et al., 2024)	viewer–film interaction	emotion	+

(“+” denotes statistical significance; “-” denotes non-significance; “N/A” denotes not applicable).

## Data Availability

No new data were created or analyzed in this study.
